# Physiological Normoxia and Absence of EGF Is Required for the Long-Term Propagation of Anterior Neural Precursors from Human Pluripotent Cells

**DOI:** 10.1371/journal.pone.0085932

**Published:** 2014-01-17

**Authors:** Bilada Bilican, Matthew R. Livesey, Ghazal Haghi, Jing Qiu, Karen Burr, Rick Siller, Giles E. Hardingham, David J. A. Wyllie, Siddharthan Chandran

**Affiliations:** 1 Euan MacDonald Centre for Motor Neurone Disease Research, University of Edinburgh, Edinburgh, United Kingdom; 2 MRC Centre for Regenerative Medicine, University of Edinburgh, Edinburgh, United Kingdom; 3 Centre for Neuroregeneration, University of Edinburgh, Edinburgh, United Kingdom; 4 Centre for Integrative Physiology, University of Edinburgh, Edinburgh, United Kingdom; University of Freiburg, Germany

## Abstract

Widespread use of human pluripotent stem cells (hPSCs) to study neuronal physiology and function is hindered by the ongoing need for specialist expertise in converting hPSCs to neural precursor cells (NPCs). Here, we describe a new methodology to generate cryo-preservable hPSC-derived NPCs that retain an anterior identity and are propagatable long-term prior to terminal differentiation, thus abrogating regular *de novo* neuralization. Key to achieving passagable NPCs without loss of identity is the combination of both absence of EGF and propagation in physiological levels (3%) of O_2_. NPCs generated in this way display a stable long-term anterior forebrain identity and importantly retain developmental competence to patterning signals. Moreover, compared to NPCs maintained at ambient O_2_ (21%), they exhibit enhanced uniformity and speed of functional maturation, yielding both deep and upper layer cortical excitatory neurons. These neurons display multiple attributes including the capability to form functional synapses and undergo activity-dependent gene regulation. The platform described achieves long-term maintenance of anterior neural precursors that can give rise to forebrain neurones in abundance, enabling standardised functional studies of neural stem cell maintenance, lineage choice and neuronal functional maturation for neurodevelopmental research and disease-modelling.

## Introduction

Recent advances in human pluripotent stem cell (hPSC) research is rapidly leading to the development of humanised cell culture models of developmental and degenerative neurological disorders. Although several neural conversion methods are available to generate neural precursors (NPCs) from hPSCs, many existing protocols describing long-term propagation result in the deregulation of spatial identity and differentiation potential [1], [2]. Efficient neural conversion of human PSCs, that mimics default mammalian neurogenesis, in defined conditions that limit extrinsic signaling cues is well established [1]–[5]. Human PSCs undergo distinct formation of radially organized columnar neuroepithelia called ‘neural rosettes’ during neural conversion from the pluripotent state [6]. These neural rosettes assume an obligate primitive anterior identity by default in chemically-defined medium (CDM) [7] and can give rise to glutamatergic forebrain neurons with dorsal telencephalic identity in the absence of known morphogens [8], [9]. Building on these observations a variety of methods are developed to generate cortical neurons, all of which notably require *de novo* neural conversion of hPSCs [10]–[14]. However, the ability to derive and, critically, maintain long-term human NPCs of anterior identity that predictably generate physiologically functional cortical neurons has not been reported.

To circumvent the need for *de novo* neural differentiation for every new experiment a number of attempts have been made to capture and propagate defined neural precursor populations, mostly relying on epidermal growth factor (EGF) and fibroblast growth factor (FGF) as mitogens [15], [16]. Even though these methods provide a homogenous neurogenic precursor population, the initial spatial identity remains subject to deregulation in long-term culture. For instance, long-term self-renewing rosette-type human embryonic stem derived neural stem cells (lt-hESNSCs) established from neural rosettes with expansion in FGF2 and EGF lose anterior identity, marked by the loss of *OTX2* expression that is required for the specification of neural precursors to become telencephalic, and predominantly differentiate into GABAergic neurons [16], [17]. In the embryonic mouse telencephalon FGF-responsive multi-potential, self-renewing NSCs emerge before EGF-responsive NSCs and distinct populations proliferate in response to these mitogens [18]. However, whether combined EGF and FGF2 treatment is permissive for the propagation of human PSC derived NPCs with anterior identity is not established.

Noting the importance of physiologically-relevant, low-O_2_ levels (3%) for embryogenesis, particularly regulating stem cell survival, fate, proliferation, genomic stability and differentiation [19]–[23] we have previously shown that NPCs can be derived *de novo* from human embryonic stem cells (hESC) in CDM at 3% O_2_ that show tri-lineage differentiation potential and are responsive to patterning cues [24].

Here, we address the combined effects of EGF signaling and O_2_ tension on long-term stability and identity of human NPCs isolated from neural rosettes and report the derivation of anterior NPCs (aNPCs) that can be propagated long-term as a monolayer and cryo-preserved, thus eliminating the need for *de novo* neural conversion from PSCs. Critically, long-term propagation of aNPCs relies on physiological O_2_ levels (3%) for culture stability and the absence of EGF for the maintenance of anterior identity. aNPCs derived at 3% O_2_ give rise to both deep- and upper-layer cortical excitatory neurons upon terminal differentiation and also retain responsiveness to developmental patterning cues. Furthermore, compared to NPCs differentiated at ambient O_2_ (21%), neurons derived from aNPCs at physiological O_2_ levels display enhanced uniformity and speed of functional maturation. These findings enable reliable generation of scalable and stable NPCs with anterior identity from human PSCs that predictably generate phenotype-relevant functional neuronal subtypes necessary for modeling developmental and neurological disorders.

## Materials and Methods

### Generation and Cryopreservation of aNPCs

hESC line H9 was obtained from WiCell (Madison, WI) under full ethical/IRB approval of the University of Edinburgh. iPSC lines used in this study were either previously described [25] or derived with the ethics permission obtained from the NHS Lothian Research Ethics Committee (REC/10/S1103/10). Written informed consent was obtained from each individual participant. aNPCs were subsequently derived from parental pluripotent stem cell lines. Human ESCs and iPSCs (reprogrammed with pseudotyped retroviral vectors expressing coding sequences of genes *OCT4*, *c-MYC*, *SOX2*, *KLF4*) were maintained on CF-1 irradiated mouse embryonic fibroblasts, with Advanced DMEM/F12 (A-DMEM/F12), 20% Knockout Serum Replacement, 10 ng/mL basic FGF2, 1 mM L-glutamine, 100 mM 2-mercaptoethanol and 1% penicillin/streptomycin (P/S). Human PSCs were neurally converted in suspension in CDM as described in [26]. The media was changed to Base media (A-DMEM/F12, 1% P/S, 1% Glutamax, 1% N2), 0.4% B27, 2.5 ng/mL FGF2 upon observation of radially organised structures in neurospheres (10–21 days) and plated on Laminin (Sigma) coated tissue culture plates (Nunc) a week later. Neural rosettes were mechanically isolated, dissociated with Accutase (Sigma) and 20–40 k cells were plated in one Laminin-coated well of a 96-well plate in proliferation media (Base media, 0.1% B27, 10 ng/mL FGF2 and 10 ng/mL EGF where stated). aNPCs were grown to high density before passaging 1∶2 with Accutase on laminin coated plates until passage 5–6 and maintained on 1∶100 Reduced-growth factor Matrigel (BD Biosciences) coated plates thereafter.

For cryopreservation, aNPCs were made into a single cell suspension with Accutase (Sigma), spun down and 1×10^6^ cells/cryovial were re-suspended in proliferation media supplemented with 10% DMSO (Sigma). Cryovials were placed in a CoolCell® Cell Freezing Container (Biocision), put into a −80°C freezer over night and transferred to liquid nitrogen the following day for long term storage. For defrosting stored aNPCs, one cryovial was thawed out at 37°C in a water bath, cells were re-suspended in 10 mls of proliferation media and spun down. The cell pellet was then re-suspended in 1 ml of proliferation media and plated in one well of a 12-well tissue culture plate coated with 1∶100 Reduced-growth factor Matrigel (BD Biosciences).

### Differentiation of aNPCs

aNPCs were plated in default media (A-DMEM/F12, 1% P/S, 0.5% Glutamax, 0.5% N2, 0.2% B27, 2 µg/mL Heparin (Sigma)) on poly-D-lysine (Sigma), laminin (Sigma), fibronectin (Sigma) and matrigel coated coverslips for differentiation and fed twice a week. Default media was supplemented with 10 µM forskolin (Tocris) in weeks 2 and 3. From week 4 onwards forskolin was removed and default media was supplemented with 5 ng/mL BDNF and 5 ng/mL GDNF.

For motor neuron differentiation aNPCs were first caudalised in proliferation media with 0.3 µM RA and then plated down on poly-D-lysine (Sigma), laminin (Sigma) and fibronectin (Sigma) coated coverslips in Neurobasal, 0.1 µM RA, 2 µM Purmorphamine (Calbiochem), 1% N2, 1% P/S, 1% Glutamax, 5 ng/ml bFGF for 7–10 days to generate motor neuron presurcors. Motor neuron precursors were replated and media was gradually switched to Neurobasal, 0.5% N2, 0.2% B27, 1% P/S, 0.5% Glutamax, 10 ng/ml BDNF, 10 ng/ml GDNF.

### PCR & qRT-PCR

RNA was isolated using the RNeasy kit (Qiagen) and genomic DNA was removed with the DNA-Free kit (Ambion). cDNA was synthesised using 0.5 µg total RNA with the DyNAmo™ cDNA Synthesis Kit (Thermo). Technical replicates as well as no template and no RT negative controls were included and at least three biological replicates were studied in each case. Real-time quantitative PCR reactions were set up with DyNAmo ColorFlash SYBR Green qPCR kit (Thermo) or TaqMan Universal PCR Master Mix (Applied Biosystems) and run on a CFX96 System (BioRad) or 7300 Real Time PCR System (Applied Biosystems). The data were analysed using the iCycler software (BioRad) or the MxPro QPCR analysis software (Stratagene) and the qbase PLUS software (Biogazelle) for statistical comparisons. Primer sequences are provided in Table S1. Human foetal brain total RNA (21 weeks old) sample was purchased from Stratagene. *BDNF* induction was assessed by qRT-PCR after membrane depolarization of five week old neurons with 25 mM KCl and 5 µM FPL 64176 (Sigma) treatment in full media for four hours. Primer sequences are given in Table S1.

### Immunofluorescence

Cells were fixed with 4% PFA (+0.1% glutaraldehyde for Reelin staining), permeabilised with 0.2% Triton X-100 at room temperature, and then blocked in 3% goat or donkey serum, followed by incubation with primary (Table S2) and secondary antibodies (Alexa Fluors, Invitrogen). The nuclei were counterstained with 4′,6-diamidino-2-phenylindole (DAPI, Sigma) and coverslips were mounted on slides with Fluorsave (Merck). Cells were imaged using an Axioscope (Zeiss) or Observer (Zeiss) miscroscope and the images were processed and using Axiovision v. 4.8.1 (Zeiss). Fields based on uniform DAPI staining were selected and imaged in four channels for cell counts between weeks 3–5. On average more than 400 cells were analysed for each marker from at least three independent experiments. Cryosectioning of neurospheres was performed as previously described [26].

### Karyotyping

Standard G-banding chromosome analysis was performed by the Addenbrooke’s Hospital, Cytogenetics Unit, Cambridge, UK.

### Calcium Imaging

Ca^2+^ imaging was performed as described [27] at 37°C in aCSF (in mM; 150 NaCl, 3 KCl, 10 HEPES, 2 CaCl_2_, 1 MgCl_2_, 1 glucose, pH 7.2). Briefly, cells were loaded with Fluo-3 AM (11 µM; from a stock solution of Fluo-3 (2.2 mM) dissolved in anhydrous DMSO containing 20% (w/v) Pluronic detergent) for 30 min at 37°C. Fluo-3 fluorescence images (excitation 472±15 nm, emission 520±15 nm) were taken at one frame per 5 s using a Leica AF6000 LX imaging system, with a DFC350 FX digital camera. Cells were depolarized using an elevated K^+^ solution (in mM; HEPES 10, KCl 170, MgCl_2_ 1 and CaCl_2_ 2, pH 7.2) added to the medium to acheive a final [K^+^] of 50 mM) and the L-type VGCC agonist FPL 64176 (5 µM). To calibrate images, Fluo-3 was saturated by adding ionomycin (50 µM) to the perfusion chamber (to obtain F_max_) and quenched with MnCl_2_ (10 mM)+ionomycin (50 µM) to levels corresponding to 100 nM Ca^2+^ [28], which was in turn used to calculate F_min_. Free Ca^2+^ concentrations were calculated from fluorescence signal (F) according to the equation [Ca^2+^] = Kd(F – F_min_)/(F_max_ – F), and expressed as a multiple of the Kd of Fluo-3 (which is approximately 315 nM). Approximately 350 cells were analysed within 7 independent experiments.

### Electrophysiology

The whole-cell patch-clamp configuration was used to record macroscopic currents from human ES-cell derived cortical neurones using an Axon Multiclamp 700B amplifier (Molecular Devices, Union City, CA). Patch electrodes were filled with a solution comprising (in mM): K-gluconate 155, MgCl_2_ 2, Na-HEPES 10, Na-PiCreatine 10, Mg_2_-ATP 2 and Na_3_-GTP 0.3, pH 7.3 (300 mOsm) and possessed resistances of 4–7 MΩ. Coverslips containing cultured cortical neurones were placed in the recording chamber, which was super-fused with an extracellular solution composed of (in mM) NaCl 152, KCl 2.8, HEPES 10, CaCl_2_ 2, glucose 10, pH 7.3 (320–330 mOsm) using a gravity-feed system at room temperature (20–23°C). The liquid junction potential was calculated to be +14 mV (JPCalc, Clampex). Current-clamp recordings were performed at potential of –74 mV in the presence of CNQX (5 µM), D-APV (50 µM), PTX (50 µM) and strychnine (20 µM) with bridge balance mode with pipette capacitance neutralised. Individual experimental designs for electrophysiological assessement i.e., holding potential, pharmacology and voltage-ramp protocols, are detailed within the figure legends and Text S1. For all recordings, holding currents were less than –100 pA, series resistance was no more than 35 MΩ, and experiments where *R_s_* drifted more than 20% of starting value were terminated. Series resistance was compensated by 80% in the voltage-clamp configuration. Current and voltage measurements were low-pass filtered online at 2 kHz, digitised at 10 kHz *via* a BNC-2090A (National Instruments) interface, and recorded to computer using the WinEDR V2 7.6 Electrophysiology Data Recorder (J. Dempster, Department of Physiology and Pharmacology, University of Strathclyde, UK; www.strath.ac.uk/Departments/PhysPharm/).

### Statistical Analysis

Data are presented as mean ± s.e.m. Statistical analysis for parametric data was conducted using, as appropriate, unpaired t-test and one-way analysis of variance (ANOVA) with the *post hoc* Tukey’s test. For non-parametric data, ANOVA analysis was performed using the Kruskal-Wallis test with the *post hoc* Dunn’s test.

## Results

### EGF Signaling Deregulates Human NPC Anterior Identity

**Figure 1:**
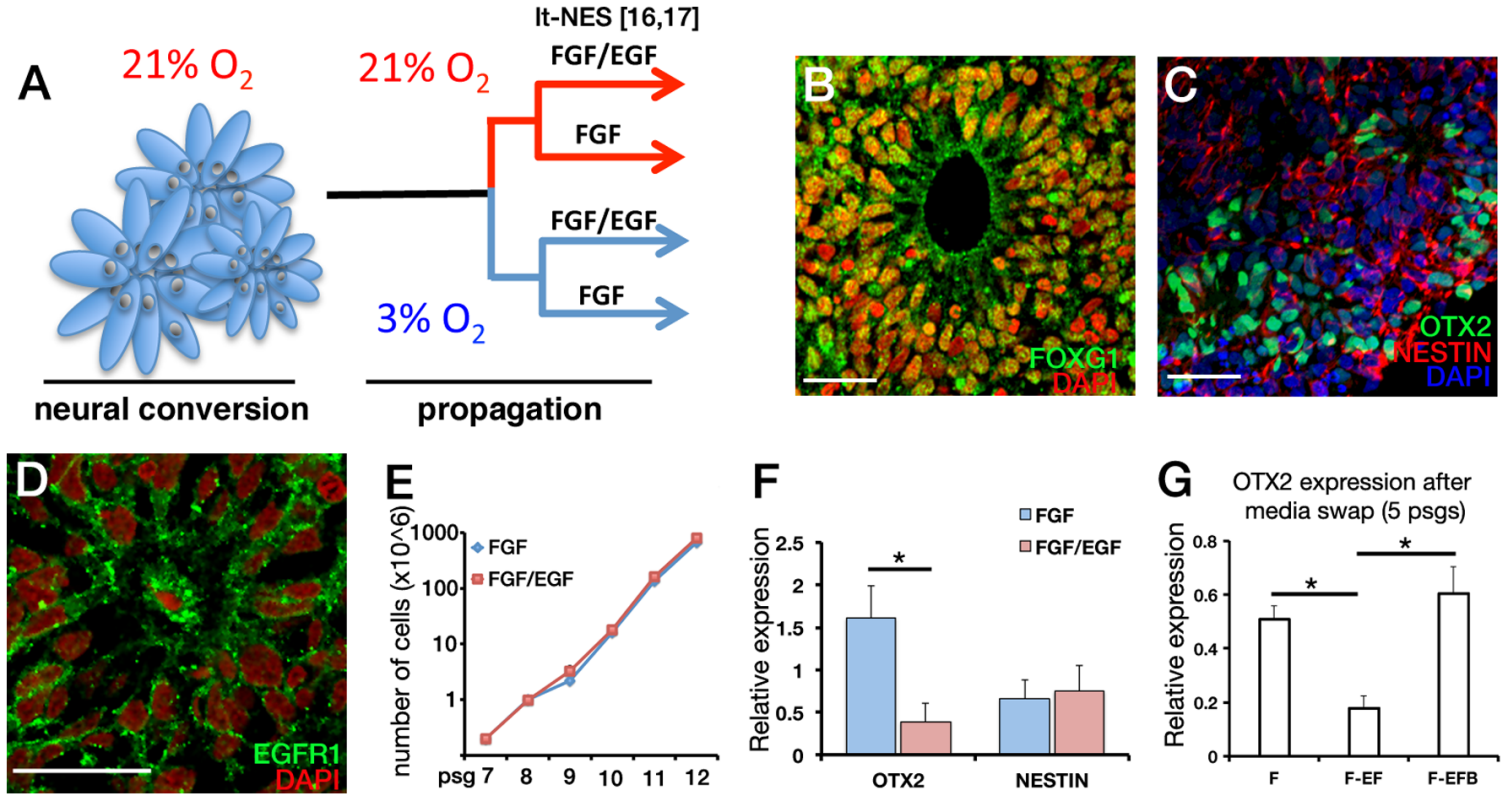
EGF signaling deregulates human anterior NPC identity. (**A**)**:** Schematic of the experimental outline. Human PSCs were neuralised at 21% O_2_ and dissociated neural rosettes were propagated at 3% or 21% O_2_ with mitogens as shown. EGF/FGF propagation at 21% O_2_ was described previously [16], [17]. (**B–D**)**:** Immunofluorescence analysis of neurosphere cryosections before platedown. Radially organised neuroepithelia express FOXG1, OTX2 and NESTIN, and display uniform staining of EGFR1. Scale bars are 20 µm (**E**)**:** aNPCs maintained in FGF2 or EGF/FGF2 containing media proliferate at similar rates (n = 3, cumulative cell count from 5 passages shown). (**F**)**:** Propagation of aNPCs in EGF/FGF2 results in down regulation of anterior marker *OTX2* by passage15, while the expression of neural progenitor marker *NESTIN* remains unchanged as determined by qRT-PCR relative expression analysis, n = 4, *P*<0.05, un-paired *t*-test. (**G**)**:** *OTX2* relative expression analysis by qRT-PCR of aNPC cultures established in FGF2 (F) and propagated in EGF/FGF2 (E/F) or EGF/FGF2/EGFR-blocker (E/F/PD) for five passages. Relative *OTX2* expression is significantly down-regulated in E/F cultures compared to F and E/F/PD, n = 4, *P*<0.05, ordinary ANOVA with Tukey’s multiple comparison test.

To address the effects of mitogens FGF2 and EGF (Fig. 1A) on the regional identity of long-term propagated NPCs, studies were first undertaken on the H9 hESC line and subsequently replicated on multiple independently derived human induced PSC lines (hiPSCs). H9 hESCs were first neurally converted under substrate-free conditions in CDM at 21% O_2_ as described previously [24], kept in suspension culture until internal neural-rosette like structures began to form from 7–14 days in culture and were either processed for cryosectioning to determine cellular composition or plated onto laminin-coated substrate prior to mechanical isolation of neural rosettes (Fig. S1A). Immunofluorescence analysis of these 3D neural aggregates revealed radially organized cells around a central lumen that were positive for the telencephalic transcription factor FOXG1 [29] in agreement with previous studies showing self-organisation of cortical tissues from hPSCs (Fig. 1B) [11], [13], [16]. Cells within the radially-organised structures also expressed early neuroepithelial markers PAX6, NESTIN and OTX2, a homeodomain transcription factor expressed in the anterior neuroectoderm [30] (Fig. 1C and Fig. S1B).

Propagation of neural rosette-derived NPCs in the presence of EGF and FGF2 at 21% O_2_ has been previously reported, termed long-term self-renewing neuroepithelial-like stem cells (lt-NES cells) [16], [17]. Lt-NES cells in culture progressively loose the initial anterior identity marked by loss of OTX2 expression, assume a transcriptional factor expression profile consistent with hindbrain identity and generate predominantly GABAergic neurons upon differentiation. Therefore, EGF and FGF2 propagation of rosette-derived NPCs at 21% O_2_ was not investigated further in this study. To address the effect of EGF and FGF signaling on aNPCs propagated at 3% O_2_, noting widespread expression of EGFR on neural-rosette like columnar cells (Fig. 1D), isolated neural-rosettes were enzymatically dissociated and cultured either in FGF2 alone or EGF/FGF2 containing conditions. No difference in growth curves of aNPCs propagated in FGF2 or EGF/FGF2 was found (Fig. 1E), but aNPCs maintained in EGF/FGF2 significantly down regulated *OTX2* by passage 15, whilst the levels of neural progenitor marker *NESTIN* were unaffected (Fig. 1F). To confirm that deregulation of anterior identity was dependent on EGF signaling, early passage aNPCs established with FGF2 were swapped into EGF/FGF2 or EGF/FGF2 treatment with a selective inhibitor of EGFR tyrosine kinase activity PD168393 (EGF/FGF2/PD) and propagated for five passages. Analysis by qRT-PCR revealed that FGF2 or EGF/FGF2/PD treatment distinctly maintained *OTX2* expression in sharp contrast to EGF/FGF2 treated cultures (Fig. 1G).

### FGF2-propagated aNPCs Maintain a Long-term Anterior Identity When Propagated in 3% O_2_

**Figure 2:**
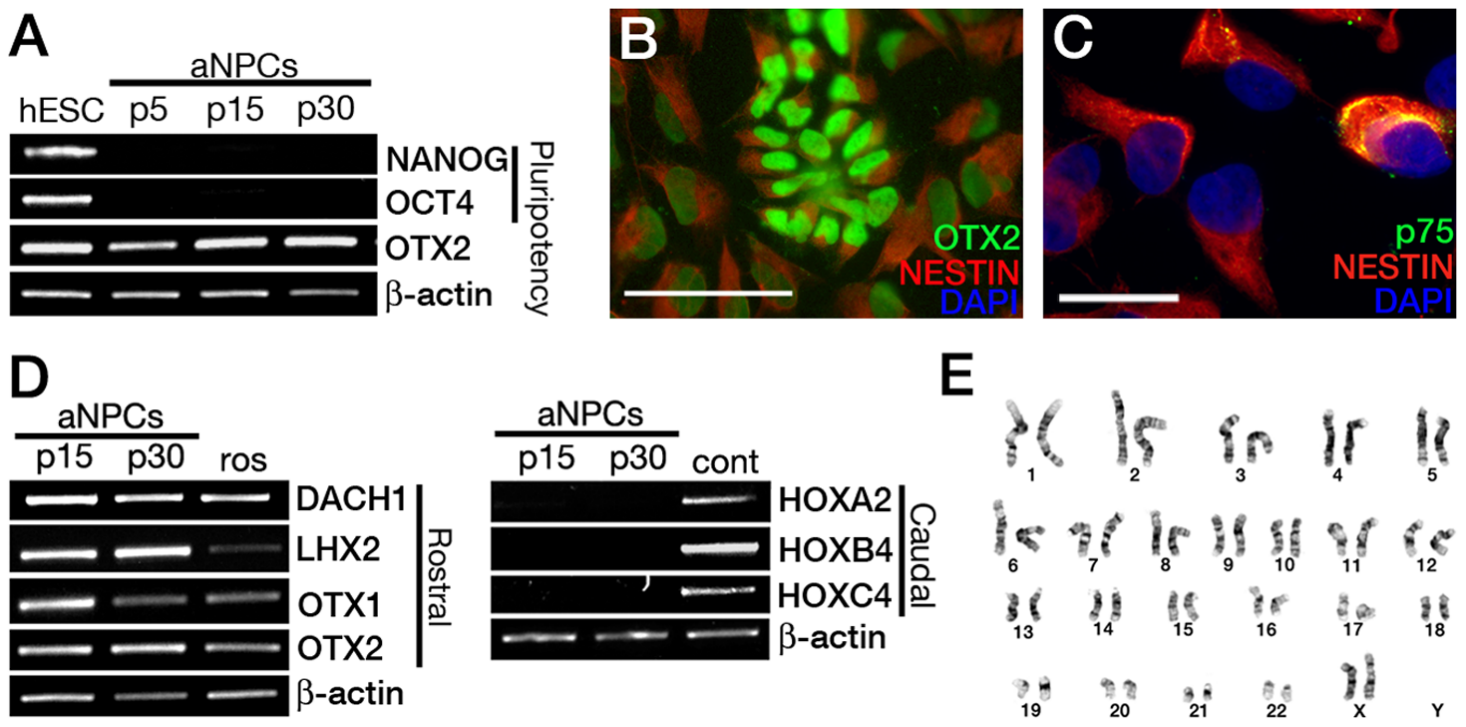
Characterisation of aNPCs in long-term culture. (**A**)**:** Expression of pluripotency markers *NANOG* and *OCT4* are not detectable by RT-PCR in 3% O_2_ aNPCs while anterior neuroectoderm marker *OTX2* expression is maintained (p:passage). (**B**)**:** Proliferating aNPCs display uniform NESTIN expression and mosaic OTX2 expression (scale bar 50 µm). (**C**)**:** Immunohistochemical staining against p75 (green), NESTIN (red) and DNA (blue) in proliferating 3% O_2_ aNPCs (scale bar 20 µm). (**D**)**:** RT-PCR analysis of rostral markers *DACH1*, *LHX2*, *OTX1*, *OTX2* and caudal markers *HOXA2*, *HOXB4* and *HOXC4* in passage 15 and 30 aNPCs maintained in FGF compared to isolated neural-rosettes (Ros) or RA-patterned aNPCs (cont), respectively. (**E**)**:** Representative chromosome analysis of a H9 hESC-derived aNPC line (passage 25) by G-banding showed that long-term propagating NPCs maintained a normal karyotype.

aNPCs established in the presence of FGF2 showed complete loss of pluripotency markers *NANOG* and *OCT4* by passage 5 and continued to express high levels of *OTX2* up to passage 30 (Fig. 2A). aNPCs propagated long-term as a monolayer displayed rosette-like patterns in culture and quantitative immunofluorescence analysis revealed that proliferating cells uniformly expressed NESTIN with high and low levels of OTX2 expression (84.6±6.4%; n = 4 independent derivations, passage >20; regardless of expression levels) (Fig. 2B). Neural crest contamination of propagating aNPC cultures was also minimal as determined by p75 immunofluorescence (3.8±0.8%; n = 4 independent derivations, passage >20; Fig. 2C). Anterior NPCs also maintain the expression of rostral/telencephalic markers *OTX1*, *OTX2*, *LHX2* and *DACH1* without up regulation of caudal markers *HOXA2*, *HOXB4* and *HOXC4* (Fig. 2D) and maintain a stable karyotype (Fig. 2E). Collectively these findings show that FGF2 alone at 3% O_2_ supports long-term maintenance of human PSC-derived NPCs with a stable anterior identity.

### FGF2-derived Early Passage aNPCs Propagated in Either 21% or 3% O_2_ have Comparable Regional Identity but give rise to Neurons with Different Functional Potential

**Figure 3:**
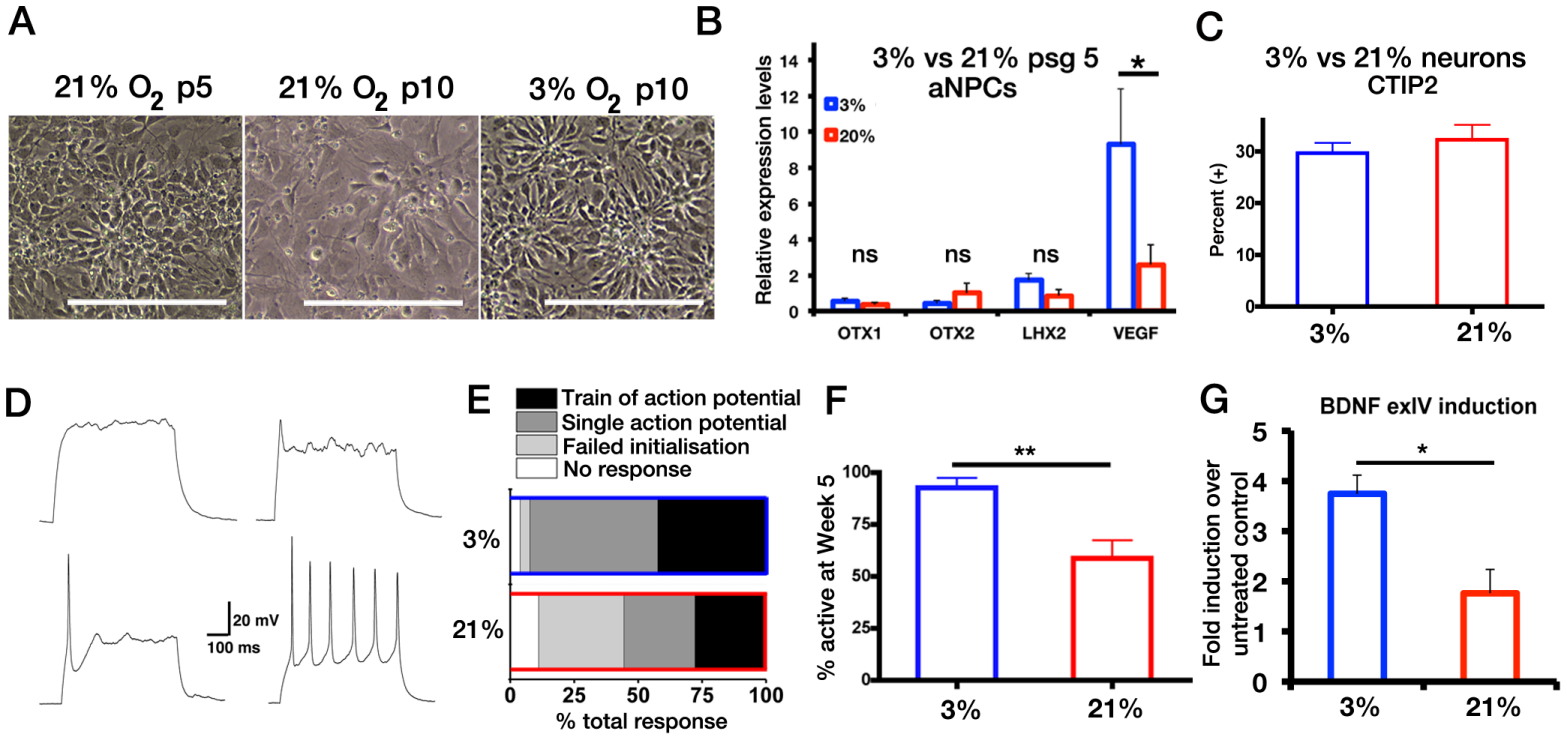
Cortical neurons derived at 3% O_2_ display uniform and enhanced functional maturation. (**A**)**:** Phase-contrast images of aNPCs derived from the same rosette-isolation, propagated in FGF2 at 21% and 3%. aNPCs proliferated at 21% O_2_ with FGF2 show rosette-like morphology at early passages (21% O_2_ p5) but display increased differentiation and altered morphology with successive passaging (21% O_2_ p10). aNPCs propagated at 3% O2 with FGF2 show stable cellular morphology (3% O_2_ p10). Scale bars 200 µm. (**B**)**:** Comparison of *OTX1*, *OTX2*, *LHX2* and *VEGF* relative expression levels between 3%- and 21%-O_2_ derived aNPCs at passage 5 as determined by qRT-PCR, n = 4, * P<0.05, ns: non-significant, un-paired *t*-test. (**C**)**:** Quantitative immunohistochemical analysis of CTIP2 expression after four weeks of differentiation of aNPCs derived at 3%- and 21%-O_2_ at passage 5. (**D**)**:** Example current-clamp recordings of activity induced by a depolarising current pulse (+30 pA) from a potential of –74 mV. From *left* to *right*, the categorised responses depict; (top panels) *no response*, *failed initialisation*; (bottom panels) *single AP*, *train of APs*. (**E**)**:** Bar graph showing the cumulative distribution of activity response of 3%- and 21%-O_2_ aNPC-derived week 5 neurons from three independent *de novo* aNPC derivations. (**F**)**:** Bar graph summarising mean (± s.e.m.) percentage of active 3% and 21% O_2_ aNPC-derived week 5 neurons per *de novo* batch of aNPC paired derivations (n = 3 batches; *P*<0.05; unpaired *t*-test). Mean input resistance measurements were not different between conditions, but a difference (p<0.05) in whole-cell capacitance was observed (21%: 12.8 pF vs 3%: 16.3 pF). (**G**)**:** Comparison of BDNF exon IV transcription induction between 5 week old neurons differentiated at 3%- and 21%-O_2_ in response to membrane depolarisation with K^+^ in the presence of FPL 64176 (5 µM) as determined of qRT-PCR. Expression is normalised to β-*ACTIN* and fold induction normalised to untreated respective control cultures is shown (n = 3, *P*<0.05, unpaired *t*-test).

Routinely, human PSC-derived NPCs are propagated only a few passages in order to increase their yield before differentiation. Passage 5 was chosen as FGF2-only treated aNPCs grown at 21% O_2_ displayed spontaneous differentiation, morphological changes and notable cell death beyond passage 6–7, and indeed only one of six derivations was maintained to passage 10 (Fig. 3A). For aNPCs propagated in 3% O_2_ the expression of anterior markers *OTX1*, *OTX2* and *LHX2* were comparable (Fig. 3B) however these aNPCs had significantly higher levels of *VEGF* expression as expected [23]compared to 21%. Furthermore at 4 weeks post differentiation, both 3% and 21% populations gave rise to similar proportions of neurons positive for cortical deep-layer marker, CTIP2 (29.6±1.9% *versus* 30.7±2.5%, respectively, Fig. 3C), suggesting comparable differentiation potential.

To determine whether O_2_ levels altered the functional potential of NPCs we next compared the maturation profile of paired passage 5 cultures propagated at 3% or 21% O_2_. Functional properties of 21% vs 3% O_2_-derived neurons differentiated from passage five FGF2-derived aNPCs concurrently generated from three PSC batches were investigated. The activity of week 5 neurons was assessed by their ability to fire action potentials. Neurons were classified as being ‘active’ if they were able to either fire single or trains of APs in response to depolarising current injection. For all batches, 21% O_2_-derived neurons were significantly less active than those derived in 3% O_2_ (Fig. 3D–F). Importantly, neurons differentiated from hiPSC-derived aNPCs in 3% O_2_ also showed robust AP firing (data not shown). Neuronal development and adaptive functions also require activity-dependent gene regulation and thus we investigated the transcriptional activation of the *BDNF* gene [31] by KCl-induced membrane depolarization. 3% O_2_-derived neurons had ∼2-fold higher transcriptional induction of the *BDNF* exon IV compared to 21% O_2_ counterparts (Fig. 3G). Collectively, these observations suggest that 21% O_2_ is not permissive to long-term propagation of aNPCs under current culture conditions and derivation of neurons at physiological O_2_ also provides a functional improvement compared to 21% O_2_.

### FGF2-propagated aNPCs in 3% O_2_ Generate Cortical Neurons and Retain Developmental Competence to Patterning Cues

**Figure 4:**
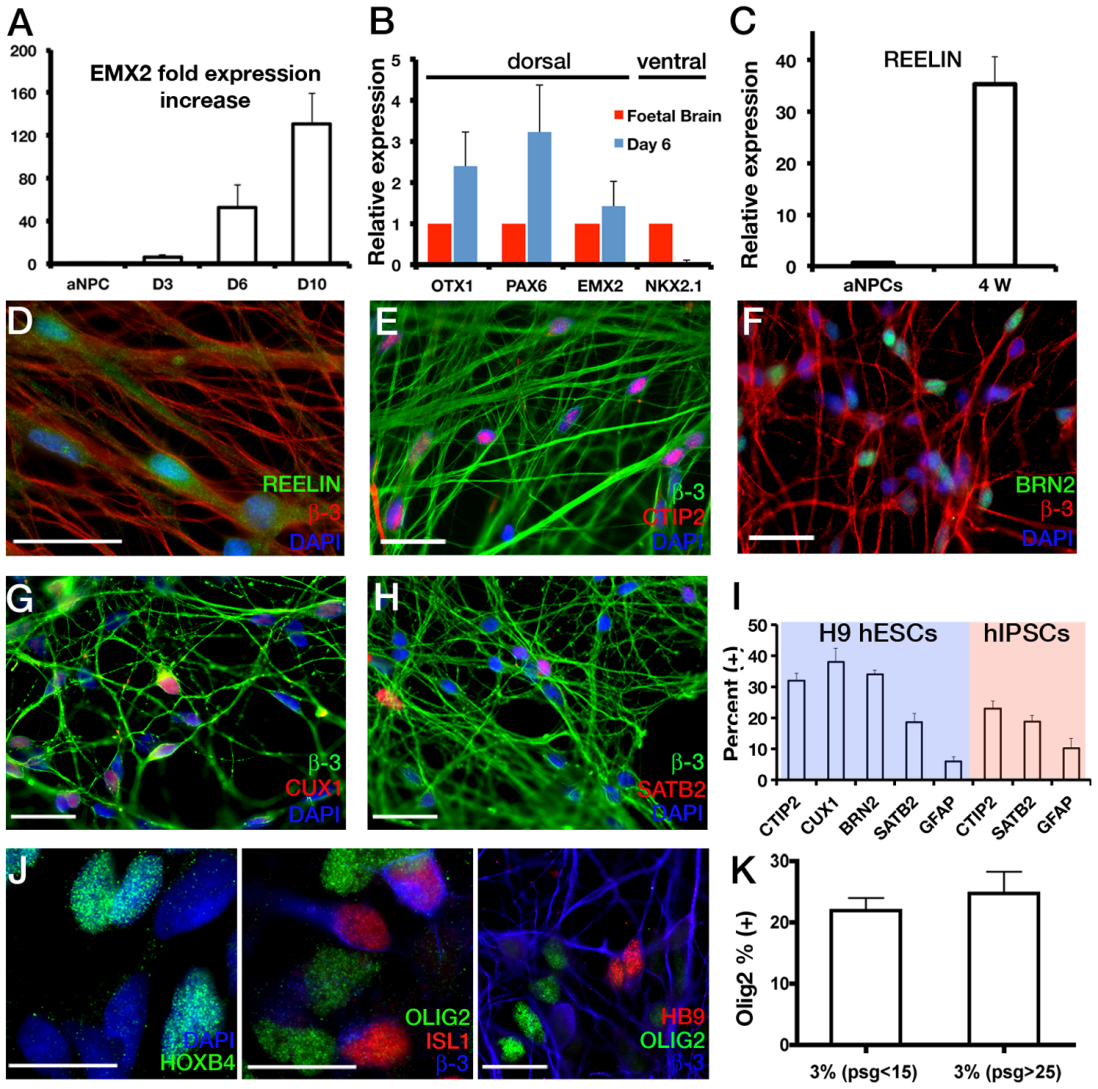
aNPCs maintained in FGF give rise to cortical neurons by default and are responsive to patterning cues. (**A**)**:** Upon withdrawal of FGF2, aNPCs display time-dependent upregulation of dorsal telencephalic marker *EMX2* as determined by qRT-PCR (D = days *in vitro* differentiation. *EMX2* expression levels are normalised to levels detected in proliferating aNPCs ( = 1), β-actin is used as housekeeping control. *EMX2* D10 expression = 130.4±29.2, n = 4. (**B**)**:** Gene expression in human fetal brain (FB) and aNPC cultures differentiated for 6 days. All expression levels are normalized to levels detected in human tissue ( = 1). Data are represented as mean ± SEM, n = 4 for differentiated aNPCs, passage >20. For tissue, n = 1. aNPC derived neurons express REELIN by 4 weeks of differentiation as determined by qRT-PCR (**C**; aNPCs vs neurons: 0.7±0.2 vs 35.3±5.4, n = 4, *P*<0.001, un-paired *t*-test) and immunofluorescence (**D**). (**I**)**:** Quantitative immunohistochemical analysis of neurons differentiated from aNPCS revealed expression of both deep-layer and upper-layer cortical neuronal markers (CTIP2, BRN2, CUX1, SATB2) (**E–H**). Data from n = 4–7 differentiation experiments from three H9 hESC- (passages >20) and three hIPSC-derived (passages 10–28) aNPC lines shown. (**J**)**:** aNPCs are responsive to patterning cues, sequentially upregulating HOXB4, OLIG2, ISL1 and HB9 expression in response to treatment with RA and SHH agonist purmorphamine during motor neuron differentiation. (**K**)**:** Quantitative immunohistochemical analysis of OLIG2 induction efficiency in early and late passage aNPCs upon treatment with RA and purmorphamine (n = 3 differentiation experiments from independent, early (<15) and late (>25) passage aNPC lines). Data are represented as mean ± s.e.m. Scale bars are 20 µm.

Having established that aNPCs propagated with FGF2 at 3% O_2_ maintain a stable anterior identity we next assessed the telencephalic marker profile of aNPC-derived neurons together with their developmental competence to patterning cues. Following withdrawal of FGF2 and subsequent culture in differentiation medium the expression of *EMX2,* a transcription factor essential for the specification of cortical neuroblasts and the formation of the dorsal telencephalon [32], was robustly upregulated in a time-dependent fashion (Fig. 4A). At day 6 of differentiation, cultures expressed high levels of dorsal telencephalic markers *OTX1*, *PAX6* and *EMX2* whereas the expression of ventral telencephalic gene *NKX2.1* was not detected (Fig. 4B), suggesting that aNPCs assume a dorsal identity by default. Over the course of 5 weeks aNPCs generated neurons that were positive for REELIN and CTIP2 [33] and upper-layer markers CUX1, BRN2 and SATB2 [34] (Fig. 4 C–H). The differentiated cultures were highly enriched for neurons (86.7±3.6%, β-3 tubulin**^+^**) with less than 10% GFAP^+^ astrocyte presence (Fig. 4I and Fig. S1C). The neuronal population displayed cortical marker expression of CTIP2^+^ 31.9±2.4%, CUX1^+^ 38±4.3%, BRN2^+^ 34±1.4%, and SATB2^+^ 18.6±2.7% (Fig. 4I; n = 3–4 independent aNPC derivations, started from cryo-preserved stocks between passages 20–30). Importantly, hiPSCs neuralised in CDM also gave rise to OTX2^+^ (71.2±4.9%) aNPCs that could be propagated long-term with FGF2 at 3%O_2_ and that differentiated to cortical neurons by default (CTIP2^+^ 22.9±2.5%, SATB2^+^ 18.9±1.9%, GFAP^+^ 10.1±3.1%, n = 3 independent iPSC lines, passages 10–28, Fig. 4I and Fig. S2 A–D).

Early rosette stage cells display a broad differentiation potential and can be patterned to generate different neuronal subtypes [3], [35]. Previous studies have shown that the developmental competence of NPCs to patterning signals is temporally determined with late or long-term propagated NPCs losing the ability to respond predictably to morphogens [1], [15], [36]. Treatment of long-term aNPCs with motor neuron-inducing signals retinoic acid (RA) and puromorphamine upregulated HOXB4 expression and resulted in sequential expression of OLIG2, ISL1 and HB9, indicative of motor neuron induction (Fig. 4J). Critically, quantitative immunofluorescence analysis revealed that early and late passage aNPCs had similar OLIG2 induction efficiencies indicating that patterning potential is maintained in long-term culture (Fig. 4K; passage 5 vs 25). Similarly, hiPSC-derived lines also displayed predictable responsiveness to patterning cues RA and puromorphamine (Fig. S2F).

### Functional Characterization of Cortical Neurons Differentiated from 3% O_2_ FGF2-propagated aNPCs

**Figure 5:**
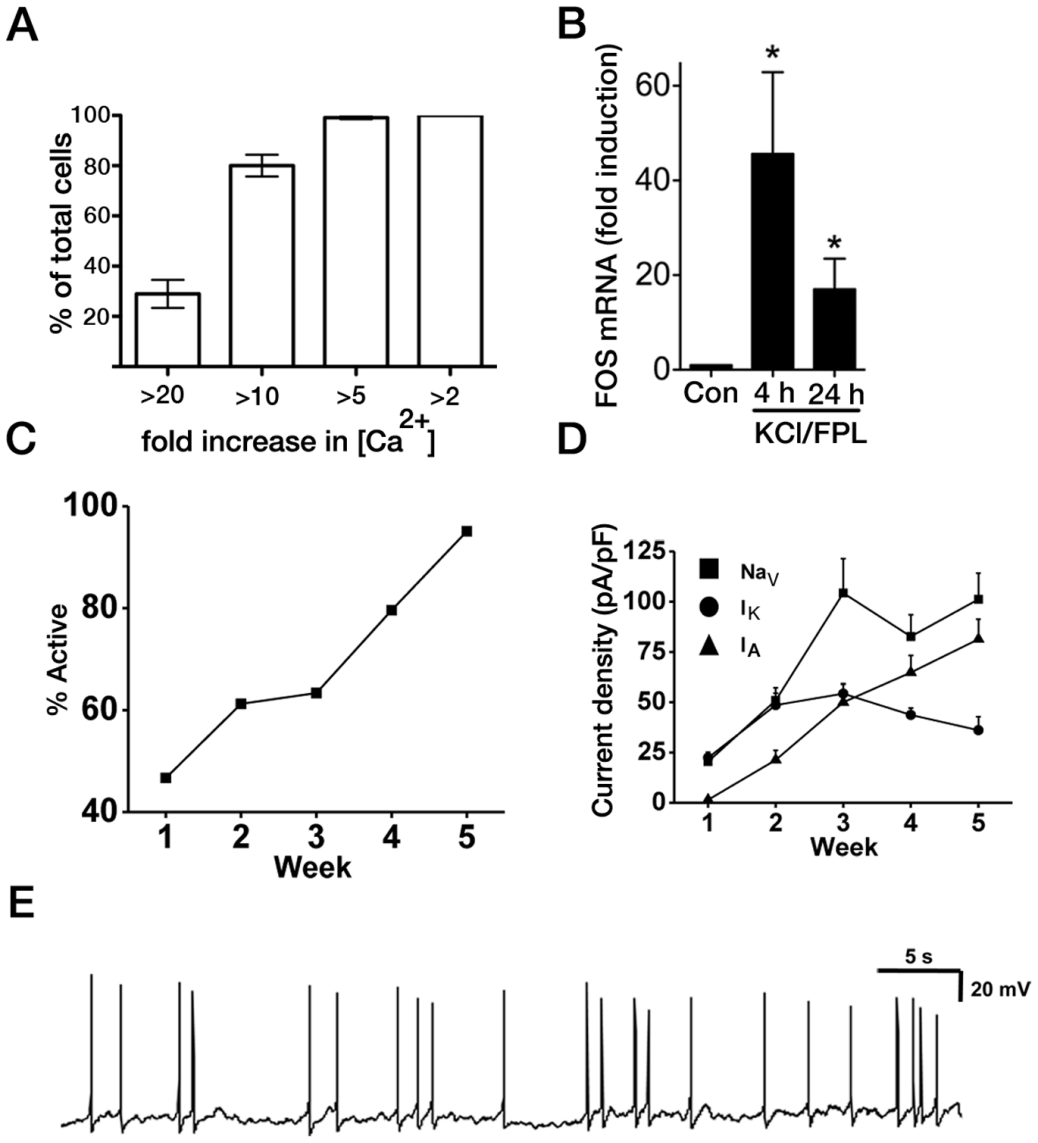
Functional characterization of cortical neurons differentiated from 3% O_2_ FGF2-propagated aNPCs. (**A**)**:** 3% O_2_ aNPC-derived neurons were subjected to Fluo-3 Ca^2+^ imaging before and during treatment with elevated K^+^ (50 mM final) in the presence of FPL 64176 (5 µM). For each cell, the fold-increase in cytoplasmic Ca^2+^ concentration was calculated (n = approximately 350 cells from n = 7 independent differentiations; passages 10–20). (**B**)**:** *FOS* mRNA fold induction in 3%-O_2_ aNPC-derived neurons in response to membrane depolarisation as determined by qRT-PCR. Expression is normalised to *GAPDH*, n = 7. (**C**)**:** Development of induced AP activity in 3% O_2_ aNPC-derived neurons over 5 weeks (n = 61 from 5 *de novo* derivations). (**D**)**:** Developmental increase in current density of voltage-gated ion channels (*Na_V_*, *I_K_*, *I_A_*) in 3% O_2_ aNPC-derived neurons. All current density values for each ion channel at week 3 are significantly higher (significance not indicated for clarity) than week 1 (*P*<0.001; Kruskal-Wallis test with *post hoc* Dunn’s test; n = 19–30 for each week, from 3 *de novo* derivations). (**E**)**:** Current-clamp recording of a 3% O_2_-derived neuron that exhibited sustained repetitive firing at a holding potential of –45 mV.

Week 5 aNPC-derived neurons were subjected to Ca^2+^ imaging before and during treatment with elevated K^+^ in the presence of the L-type voltage-gated Ca^2+^ channel agonist FPL64176 which promotes strong Ca^2+^ influx in forebrain neuronal cultures [37]. Treatment with K^+^+FPL64176 resulted in a uniformly strong increase in [Ca^2+^] (Fig. 5A and Fig. S3A) that also resulted in robust transcriptional upregulation of the immediate early gene *FOS,* the prototypical activity-dependent gene (Fig. 5B) [38].

The passive membrane properties of the 3% O_2_-derived neurons were consistent with, and indicative of, cells undergoing developmental maturation (Fig. S4 A–C). This maturing profile was confirmed by the fact that at week 5, >95% of cells fired APs (Fig. 5C) and the average number of APs spikes fired increased significantly from week 1 to week 5 (Fig. S4D). More detailed analysis was made from ‘active’ neurons and, inherently consistent with what would be expected of a population of developing cortical neurons, these cells showed expected changes in their AP firing threshold potential, AP amplitude, half-width and after-hyperpolarisation (Fig. S4 E–I). Some cells could also maintain their ability to repeatedly fire APs for more prolonged durations (Fig. 5E). AP activity and properties are ultimately determined by the co-ordinated activity of multiple voltage-gated ion channels and therefore we assessed the maturation of current density profiles of *Na_V_*, *I_K_*, *I_A_* (Fig. 5D), which showed good correlation with regard to AP development (see also Text S1 and Fig. S4 J–L). Collectively, these properties demonstrate the fidelity of the 3% O_2_ protocol to derive neurons with functionally consistent properties that are comparable with those from native cortical excitatory neurons and other human PSC-derived forebrain neurons [9], [39].

### 3% O_2_ aNPC-derived Cortical Neurons form Functional Excitatory Synapses

**Figure 6:**
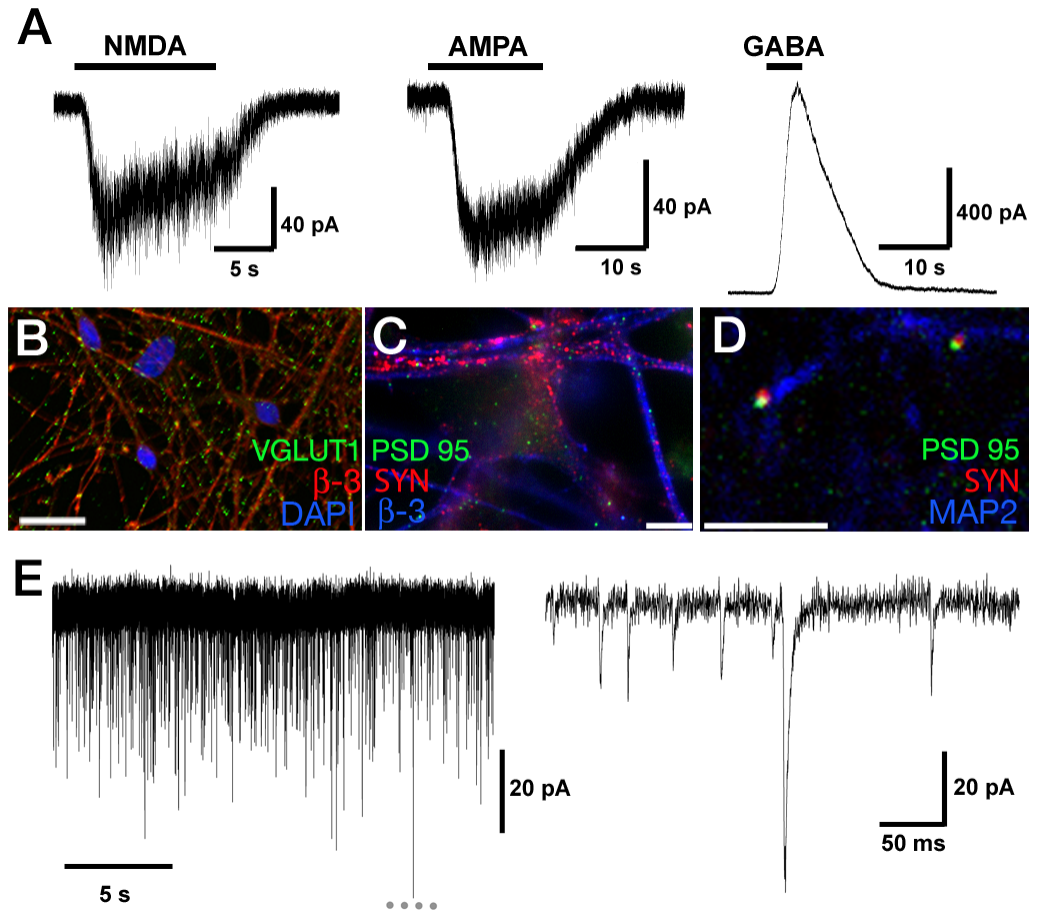
aNPCs give rise to glutamatergic neurons that can form functional excitatory synapses. (**A**)**:** Examples of whole-cell currents recorded from 3% O_2_ aNPC-derived neurons in response to bath application of NMDA (100 µM) in the presence of glycine (100 µM), AMPA (50 µM), or GABA (100 µM). Immunohistochemical staining against glutamate transporter VGLUT1 and β-3 tubulin (**B**) and post-synaptic density protein (PSD-95), Synaptophysin I (SYN) and β-3 tubulin (**C**) in aNPC-derived cortical neurons. (**D**)**:** The apposition of SYN and PSD-95 on MAP2^+^ processes (**D**) mark putative synapses. Images B and D are 0.5 µm thick single optical sections acquired by confocal microscopy. Scale bars are 20 µm. (**E**)**:** Example of miniature EPSCs recorded from a week 5 neuron held at –84 mV and recorded in the presence of TTX (300 nM), strychnine (20 µM) and picrotoxin (50 µM), plus MgCl_2_ (2 mM) to block NMDA receptor-mediated currents. All events were blocked by CNQX(5 µM).

Whole-cell voltage–clamp recordings revealed the presence of functional NMDA, AMPA, and GABA_A_ receptors in accordance with native mammalian cortical neurons (Fig. 6A). The neurotransmitter subtype identity of these aNPC-derived cortical neurons was assessed after 5 weeks of differentiation by immunofluorescence and revealed that the majority of neurites displayed extensive punctate staining for vesicular glutamate transporter 1 (VGLUT1) (Fig. 6B) but only a small fraction of neurons were positive for GABAergic interneuron marker GAD65/67 (3.5±0.4%, n = 4) (Fig. S3B), consistent with a predominant glutamatergic neurotransmitter profile. Such a VGLUT1 profile was also recapitulated in hIPSC lines (Fig. S3E). The apposition of post-synaptic density protein (PSD-95) and synaptophysin I (SYN) in processes confirmed synaptic differentiation (Fig. 6C and D). The existence of functional synapses was confirmed by the presence of AMPA receptor-mediated miniature excitatory postsynaptic currents (mEPSCs; Fig. 6E) in 40% of cells (19 from 47) albeit with variable mESPC event frequencies. This data indicate that synaptogenesis is clearly present within the culture, and at levels in accordance with other reports [9]. Collectively these data suggest that long-term propagated aNPCs generate enriched glutamatergic neuronal populations that form functional excitatory synapses. Neurons differentiated from hiPSC-derived aNPCs exhibited AMPA, NMDA and GABA-mediated currents in addition to mEPSCs (data not shown).

## Discussion

In the present study we have demonstrated that the removal of EGF and use of physiological O_2_ levels permits maintenance of NPCs with anterior identity. This enables long-term propagation of aNPCs as a monolayer that can be cryo-preserved and differentiated to highly enriched neuronal populations composed of both deep- and upper-layer cortical excitatory neurons. In addition, 3% O_2_-derived neurons display more uniform, predictable and accelerated functional development profile compared to 21% O_2_ counterparts.

We identified two factors to be critical for the maintenance of aNPCs derived from hPSCs; physiological O_2_ levels for long-term propagation and the omission of EGF to maintain anterior identity. Oxygen levels have been shown to regulate survival, proliferation and neuronal fate of both rodent and human NSCs as well as limiting their precocious differentiation [19], [21], [26], [41]. Clonal analysis of mouse cortical NSCs have revealed that culture at 21% O_2_ leads to rapid depletion of multipotential NSCs whereas expansion at 5% O_2_ permits long-term maintenance of distinct NSC populations [20]. The classical view of reactive oxygen species only in the context of cellular toxicity has recently been challenged by a number of studies demonstrating the regulation of processes such as DNA repair and NSC self-renewal by redox signaling [22], [42]. Therefore, the culture conditions for NPCs should also take into account the concept of ‘oxidative optimum’, where redox signaling present at physiological O_2_ levels is actively integrated with other intracellular signaling cascades such as PI3K/Akt in the maintenance of self-renewing NPCs [22], [42].

The derivation of self-renewing NPCs with a stable identity from hPSCs is further complicated by the heterogenous nature of NPCs that emerge from neural conversion and subsequent expansion with mitogens. EGF and FGF2 are the most frequently used growth factors for the propagation of embryonic forebrain NPCs *in vitro* [5], [15], [40], [43], [44]. The expression patterns of FGFR1 and EGFR, the key receptors for FGF and EGF signaling, in the developing rodent and human CNS germinal zones is conserved with FGFR1 being expressed earlier than EGFR in the developing telencephalon [18], [45], [46]. Several lines of evidence suggest that more than one type of neural stem cell exist in the developing cortex and these cells display differential response to FGF2 and EGF mediated signaling in a dose- and context-dependent manner. Specifically, EGF signaling can alter NSC identity and differentiation potential. For instance, over-activation of EGF signaling *in vivo* biases cortical progenitors to astrocyte lineage and reduces NSC self-renewal in the adult SVZ where as *in vitro* propagation of NSCs with EGF yields more glia than FGF [18], [47]–[52]. Accumulating evidence suggests that the homeobox gene *Otx2* lies at the intersection of pluripotent stem cell maintenance and anterior neuroectoderm commitment and differentiation. Otx2 is proposed to be an intrinsic determinant of embryonic stem cell state, is expressed in the epiblast as well as ESCs and is also required in the anterior neuroectoderm to induce telencephalic gene expression for forebrain specification [30], [53]. Independent of the method of derivation, PSC-derived NPCs propagated in EGF and FGF2 lose this inherent OTX2 expression in culture over time, consistent with the loss of anterior identity [16], [17], [54]. We have shown that NPCs derived from hPSCs express EGFR as early as neural-rosette stage and expansion in EGF leads to downregulation of *OTX2* expression over successive passages. The ability to propagate aNPCs with FGF2 only as described here should facilitate future studies to delineate the effects of different signaling pathways on neural progenitor identity and potentially enable the isolation of NPC populations with different differentiation potentials.

Anterior NPCs maintained at physiological O_2_ levels with FGF2 alone assume dorsal telencephalic identity by default upon differentiation, generating VGLUT1^+^ excitatory cortical neurons, including CTIP2^+^ layer 6–5 subcortical projection neurons [33], BRN2^+^ layer 2–4 neurons [34] and SATB2^+^ layer 2–4 callosal projection and upper-layer neurons [55], [56], in contrast to other propagated NPCs that show deregulation of positional identity and assume a GABAergic fate [15], [16], [36]. The differentiation output of 3% O_2_ propagated aNPCs is also stable through serial passaging and cryopreservation as demonstrated by comparable numbers of CTIP2^+^ neurons generated from passage 5 aNPCs and late passage (20–30) aNPCs recovered from cryopreservation (30% vs 32%, respectively). The system described here not only achieves the long-term maintenance of bankable anterior neuronal precursors from hPSCs including iPSCs but also presents a significant functional improvement over conventional methods, yielding uniformly active cortical neuronal populations that display activity-dependent gene regulation, basic neuronal physiology consistent with native cortical neurons and spontaneous synaptic activity by 5 weeks of differentiation.

The culture of neurons at ambient O_2_ levels is a significant departure from that of the brain, which range from 1–5% O_2_ [57]. Physiological O_2_ levels is shown to improve neuronal survival [24], [58]–[60] and modulate neuronal metabolism [61], [62]. Neurons are particularly vulnerable to oxidative stress due to their high metabolic rate, relatively low levels of antioxidant enzymes and being post-mitotic cells more vulnerable to accumulation of reactive oxygen species [63]. This is of particular importance as redox signalling and oxidative stress are thought to play an important part in neuronal injury in a range of developmental and neurodegenerative disorders. This platform thus provides the opportunity to investigate neuronal responses to different stimuli such as metabolic stress, activity and proteotoxicity under physiological O_2_ levels that is more representative of *in vivo* conditions using functional human neurons.

*In vitro* disease-modeling using human PSCs requires reliable and scalable generation of physiologically-relevant and functional neuronally-enriched populations both for biochemical studies and phenotypic high-throughput screens. Therefore, there is a need for establishing conditions to maintain NPCs with defined characteristics that can be differentiated to disease-relevant neuronal cell types such as cortical excitatory neurons that are affected in a number of neurodegenerative disorders that include Alzheimer’s disease and frontotemporal dementia. To date, long-term propagatable human NPC cultures from hPSCs have been derived only under a few conditions. Neural-rosette cells (R-NSCs) isolated and propagated by Elkabetz et al, (2008) in the presence of recombinant SHH and Notch agonists represent one of the earliest NPC identities with a comprehensive differentiation potential of central and peripheral nervous system cell types [2]. However, R-NSCs haven’t been studied beyond passage 5 and cortical excitatory neuronal differentiation potential is not investigated. In addition, R-NSCs require several recombinant proteins and high-density plating for stable propagation, factors which can limit the scaling-up of cultures. Lt-NES cells, neuroepithelial-like stem cells established from isolated neural rosettes with expansion in FGF2 and EGF as a monolayer, lose their initial anterior identity in prolonged culture (passage>10) along with the capacity to generate excitatory glutamatergic cortical neurons [16], [17]. Radial-glia like NS cells were also previously established from mouse ES cells and adult and foetal forebrain in adherent culture conditions with FGF2/EGF [15], [40]. It is important to note that derivation of NS cells require both EGF and FGF2; cultures established in FGF2 alone are prone to spontaneous differentiation, have heterogeneous morphology and display increased cell death [40]. In contrast, aNPCs propagated at 3% O_2_ with FGF2 as described in this study display a stable anterior identity in culture and assume a dorsal transcriptional identity upon differentiation, giving rise to uniformly active, enriched cortical excitatory neuronal populations. Hence, aNPCs present a platform that not only reduces variation and experimental noise inherent to *de novo* differentiation runs from human PSCs but is also suitable for scalable functional, biochemical and imaging-based high-throughput studies.

## Conclusion

Our report of a robust method to generate stable, scalable and cryopreservable aNPCs that reliably generates neurons with functional properties consistent with native cortical excitatory neurons establishes a platform for human neurological disease modelling.

## Supporting Information

Figure S1 **Characterisation of aNPCs.** (A) Human PSCs were neuralised at 21% O_2_ in suspension in CDM and plated down for the mechanical isolation of neural rosettes. For each experiment several neural clusters were collected, dissociated into single cells and split into different conditions for pair-wise comparison (scale bar 400 µm). (B) Radially-organised neuroepithelia express PAX6 as determined by immunofluorescence analysis of neurosphere cryosections before platedown (scale bar 20 µm). (C) aNPCs differentiated for 5 weeks contain GFAP**^+^** (red) and S100β**^+^** (red) astrocytes. β-3 tubulin immunohistochemistry is shown in green and DNA is counter-stained with DAPI (blue) (scale bars are 20 µm).
(TIF)

Figure S2 **Generation of aNPCs from human iPSCs.** (A) Neural rosettes derived from human iPSCs give rise to aNPCs that can maintain anterior marker expression *OTX1*, *OTX2* and *LHX2* in extended culture as determined by RT-PCR (p:passage). (B) Immunofluorescence analysis of OTX2 and NESTIN expression in proliferating iPSC-derived aNPCs. Immunohistochemical staining against CTIP2, SATB2, VGLUT1 and β-3 tubulin revealed that human iPSC-derived aNPCs can give rise to glutamatergic cortical neurons by default differentiation (C-E). (F) iPSC-derived aNPCs upregulate OLIG2 expression in response to patterning with RA and SHH agonist purmorphamine.
(TIF)

Figure S3 **Characterisation of 3% O_2_ aNPCs.** (A) Example experiment showing Fluo-3 Ca^2+^ imaging upon membrane depolarisation of a single 3% O_2_ derived cortical neuronal culture. The mean ± s.e.m. of cytoplasmic Ca^2+^ concentration is shown, expressed as a multiple of the Kd ((F-Fmin)/(Fmax-F), n = 50). (B) aNPC-derived cortical neuronal cultures are occasionally positive for GAD65/67**^+^** (green) neurons. β-3 tubulin immunohistochemistry is shown in red and DNA is counter-stained with DAPI (blue).
(TIF)

Figure S4 **(A–C) Mean ± s.e.m. whole-cell capacitance (** ***C_m_*** **), resting membrane potential (** ***RMP*** **) and input resistance (** ***R_IN_*** **) after differentiation of 3% O_2_ aNPC-derived neurons (** ***P*** **<0.001; Kruskal-Wallis test with** ***post hoc*** **Dunn’s test; n = 30–90 for each week, from at least 3** ***de novo*** **derivations).** (D–H) Development of AP properties of 3% O_2_ aNPC-derived neurons. Data was obtained from the minimum current injection needed to elicit an action potential (rheobase). Figures describe number of APs per 500 ms depolarising current injection, threshold of AP deflection, AP amplitude from threshold, half-width of the AP response and after hyperpolarisation (* *P*<0.05, ** *P*<0.01 and *** *P*<0.001 from week 1 data as determined by one-way ANOVA test with *post hoc* Tukey’s test or Kruskal-Wallis test with *post hoc* Dunn’s test). Cells did not exhibit strong frequency-current input relationships. (I) Representative APs taken from active week 1 and 5 neurons. (J–L) Isolation of voltage-gated ion channel currents *Na_V_* (J), *I_K_* (K) and *I_A_* (L) from week 4–5 neurons. Protocols to isolate such conductances are described in detail in Text S1. Upper traces show example currents from which respective normalised peak current-voltage plots (n = 4–9) are constructed (lower graphs).
(TIF)

Table S1 **List of primers.**
(DOCX)

Table S2 **List of antibodies.**
(DOCX)

Text S1 **Development of voltage-gated ion channel properties underlying the action potential.**
(DOCX)
