# Supplementary figures and images for: Physiological Normoxia and Absence of EGF Is Required for the Long-Term Propagation of Anterior Neural Precursors from Human Pluripotent Cells
Source: PLoS One. 2014 Jan 17;9(1):e85932. doi: 10.1371/journal.pone.0085932 (PMC3895023; doi:10.1371/journal.pone.0085932)

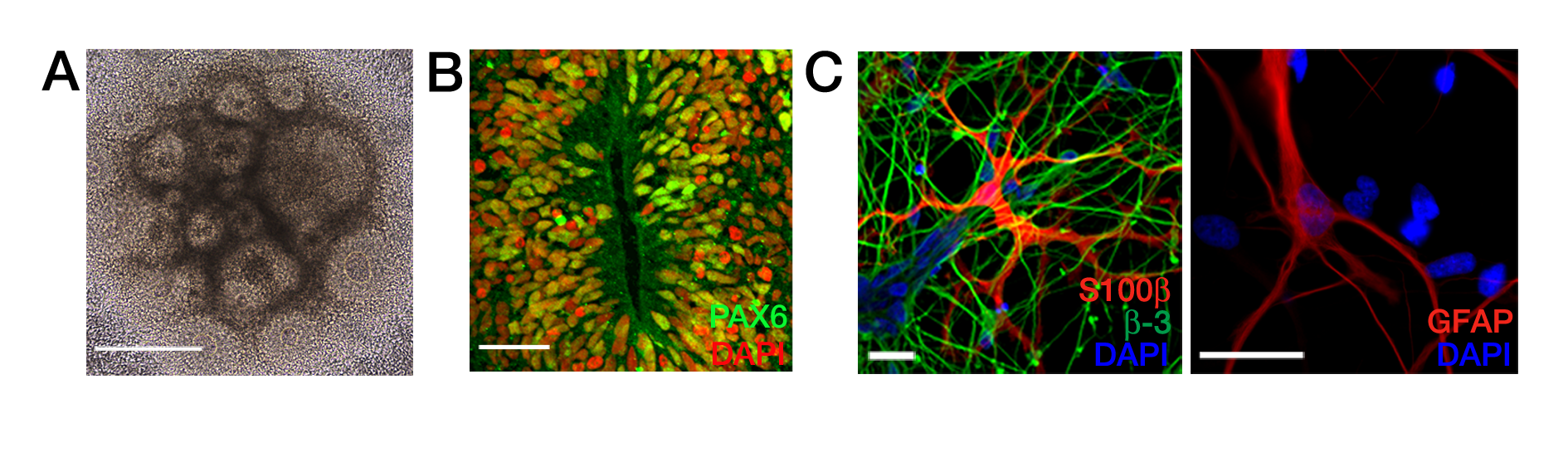

Supplement: Figure S1 — Characterisation of aNPCs. (A) Human PSCs were neuralised at 21% O2 in suspension in CDM and plated down for the mechanical isolation of neural rosettes. For each experiment several neural clusters were collected, dissociated into single cells and split into different conditions for pair-wise comparison (scale bar 400 µm). (B) Radially-organised neuroepithelia express PAX6 as determined by immunofluorescence analysis of neurosphere cryosections before platedown (scale bar 20 µm). (C) aNPCs differentiated for 5 weeks contain GFAP+ (red) and S100β+ (red) astrocytes. β-3 tubulin immunohistochemistry is shown in green and DNA is counter-stained with DAPI (blue) (scale bars are 20 µm). (TIF) [file pone.0085932.s001.tif]

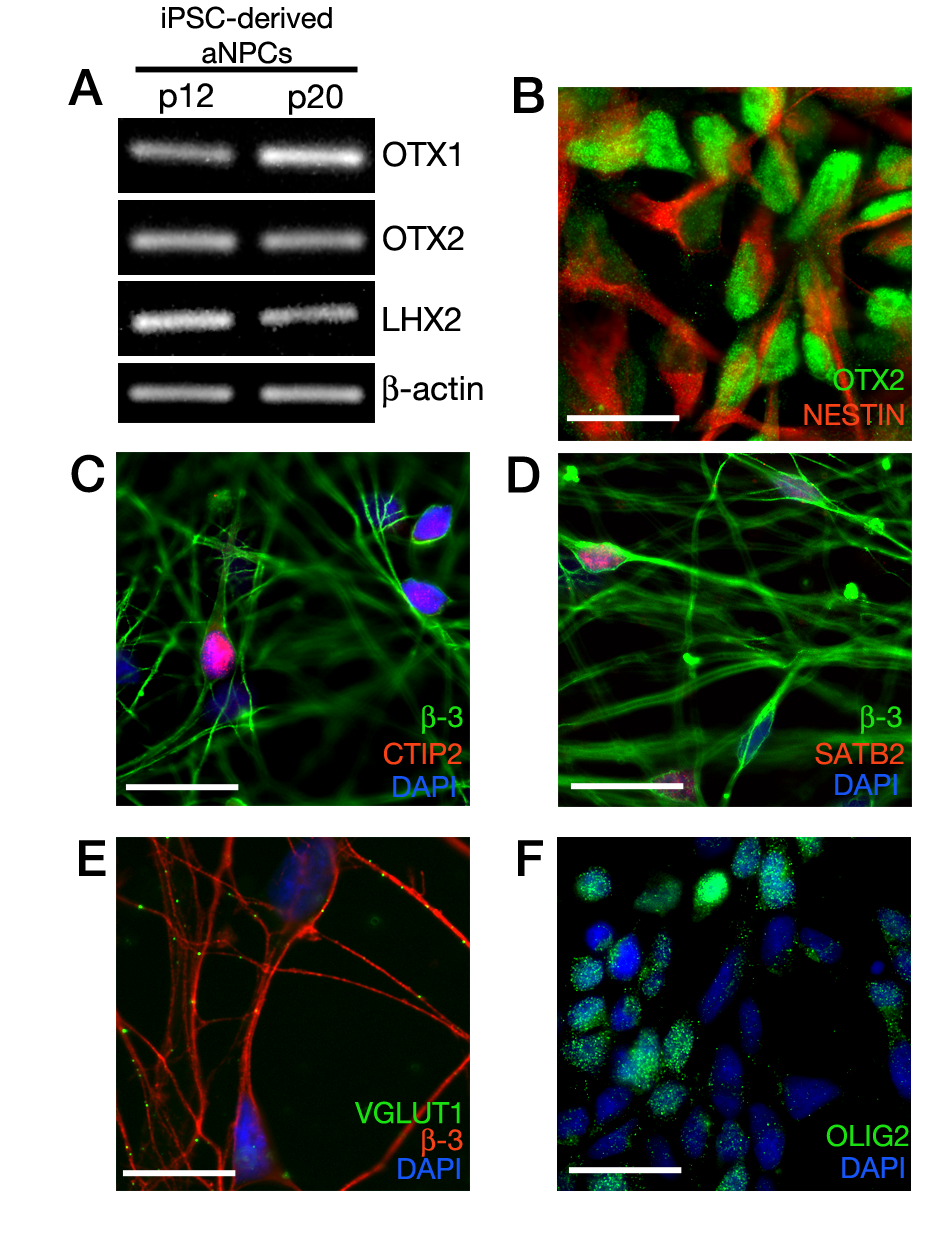

Supplement: Figure S2 — Generation of aNPCs from human iPSCs. (A) Neural rosettes derived from human iPSCs give rise to aNPCs that can maintain anterior marker expression OTX1, OTX2 and LHX2 in extended culture as determined by RT-PCR (p:passage). (B) Immunofluorescence analysis of OTX2 and NESTIN expression in proliferating iPSC-derived aNPCs. Immunohistochemical staining against CTIP2, SATB2, VGLUT1 and β-3 tubulin revealed that human iPSC-derived aNPCs can give rise to glutamatergic cortical neurons by default differentiation (C-E). (F) iPSC-derived aNPCs upregulate OLIG2 expression in response to patterning with RA and SHH agonist purmorphamine. (TIF) [file pone.0085932.s002.tif]

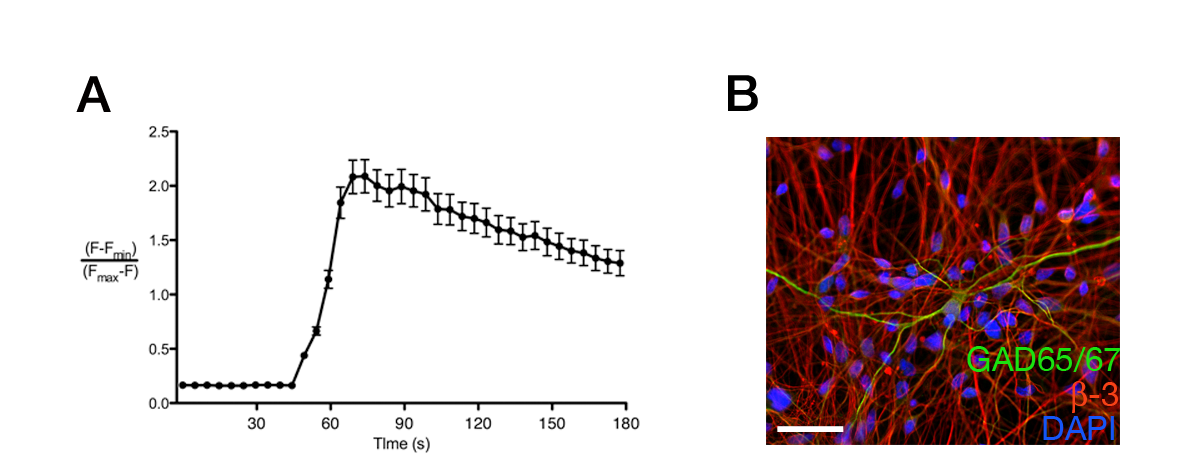

Supplement: Figure S3 — Characterisation of 3% O2 aNPCs. (A) Example experiment showing Fluo-3 Ca2+ imaging upon membrane depolarisation of a single 3% O2 derived cortical neuronal culture. The mean ± s.e.m. of cytoplasmic Ca2+ concentration is shown, expressed as a multiple of the Kd ((F-Fmin)/(Fmax-F), n = 50). (B) aNPC-derived cortical neuronal cultures are occasionally positive for GAD65/67+ (green) neurons. β-3 tubulin immunohistochemistry is shown in red and DNA is counter-stained with DAPI (blue). (TIF) [file pone.0085932.s003.tif]

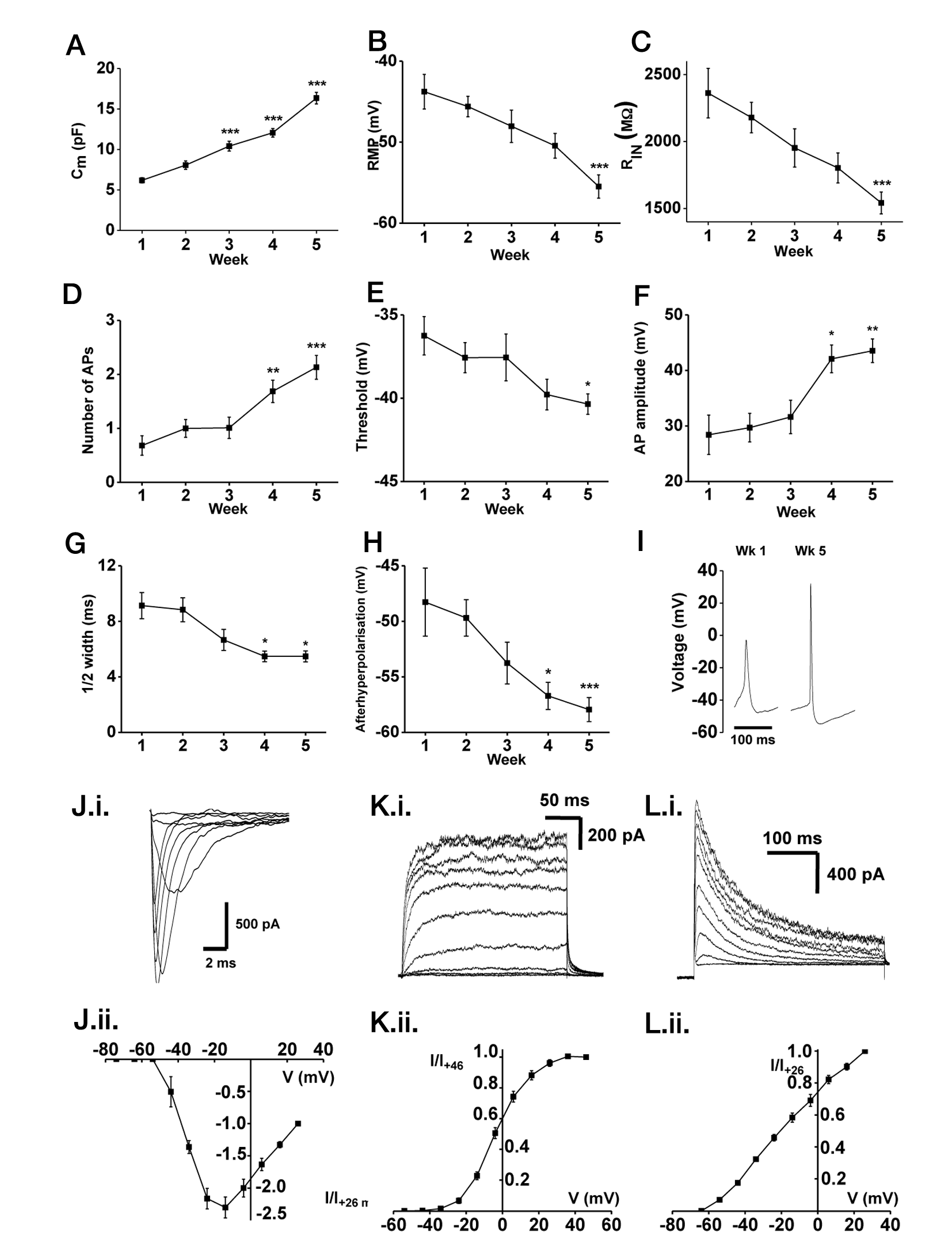

Supplement: Figure S4 — (A–C) Mean ± s.e.m. whole-cell capacitance ( Cm ), resting membrane potential ( RMP ) and input resistance ( RIN ) after differentiation of 3% O2 aNPC-derived neurons ( P <0.001; Kruskal-Wallis test with post hoc Dunn’s test; n = 30–90 for each week, from at least 3 de novo derivations). (D–H) Development of AP properties of 3% O2 aNPC-derived neurons. Data was obtained from the minimum current injection needed to elicit an action potential (rheobase). Figures describe number of APs per 500 ms depolarising current injection, threshold of AP deflection, AP amplitude from threshold, half-width of the AP response and after hyperpolarisation (* P<0.05, ** P<0.01 and *** P<0.001 from week 1 data as determined by one-way ANOVA test with post hoc Tukey’s test or Kruskal-Wallis test with post hoc Dunn’s test). Cells did not exhibit strong frequency-current input relationships. (I) Representative APs taken from active week 1 and 5 neurons. (J–L) Isolation of voltage-gated ion channel currents NaV (J), IK (K) and IA (L) from week 4–5 neurons. Protocols to isolate such conductances are described in detail in Text S1. Upper traces show example currents from which respective normalised peak current-voltage plots (n = 4–9) are constructed (lower graphs). (TIF) [file pone.0085932.s004.tif]
