# Supplementary material for: Physiological Normoxia and Absence of EGF Is Required for the Long-Term Propagation of Anterior Neural Precursors from Human Pluripotent Cells
Source: PLoS One. 2014 Jan 17;9(1):e85932. doi: 10.1371/journal.pone.0085932 (PMC3895023; doi:10.1371/journal.pone.0085932)
Supplement: Table S1 — List of primers. (DOCX) [file pone.0085932.s005.docx]

**Table S1: Primers**

| **Gene** | **Forward Primer** | **Reverse Primer** |
| --- | --- | --- |
| **BDNF Ex IV** | TCCACTATCAATAATTTAACTTC | AAACTCCCACACTCTATT |
| **c-FOS** | CTACCACTCACCCGCAGACT | AGGTCCGTGCAGAAGTCCT |
| **DACH1** | GTGGAAAACACCCCTCAGAA | CTTGTTCCACATTGCACACC |
| **EMX2** | CACAGAAACGGACAACATGG | CTTTAGACGAGGGTCGCTTG |
| **GAPDH** | cttcaccaccatggagaaggc | GGCATGGACTGTGGTCATGAG |
| **HOXA2** | TTCAGCAAAATGCCCTCTCT | TAGGCCAGCTCCACAGTTCT |
| **HOXB4** | GTGAGCACGGTAAACCCCAAT | CGAGCGGATCTTGGTGTTG |
| **HOXC4** | CGAGAAATCACAGTCGCTCTG | TGGGTAGACTATGGGTTGCTTG |
| **LHX2** | ATGCTGTTCCACAGTCTGTCG | GCATGGTCGTCTCGGTGTC |
| **NANOG** | AATACCTCAGCCTCCAGCAGATG | TGCGTCACACCATTGCTATTCTTC |
| **NESTIN** | GGCGCACCTCAAGATGTCC | CTTGGGGTCCTGAAAGCTG |
| **NKX2.1** | AACCAAGCGCATCCAATCTCAAGG | TGTGCCCAGAGTGAAGTTTGGTCT |
| **OCT4** | GTACTCCTCGGTCCCTTTCC | CAAAAACCCTGGCACAAACT |
| **OTX1** | CACTAACTGGCGTGTTTCTGC | AGGCGTGGAGCAAAATCG |
| **OTX2** | AGAGGACGACGTTCACTCG | TCGGGCAAGTTGATTTTCAGT |
| **PAX6** | ATGTGTGAGTAAAATTCTGGGCA | GCTTACAACTTCTGGAGTCGCTA |
| **β-actin** | GTTACAGGAAGTCCCTTGCCATCC | CACCTCCCCTGTGTGGACTTGGG |
