# Supplementary material for: Physiological Normoxia and Absence of EGF Is Required for the Long-Term Propagation of Anterior Neural Precursors from Human Pluripotent Cells
Source: PLoS One. 2014 Jan 17;9(1):e85932. doi: 10.1371/journal.pone.0085932 (PMC3895023; doi:10.1371/journal.pone.0085932)
Supplement: Table S2 — List of antibodies. (DOCX) [file pone.0085932.s006.docx]

**Table S2: List of antibodies used**

| **Antibody** | **Host** | **Company** |
| --- | --- | --- |
| **BRN2 (C-20)** | Goat polyclonal | Santa Cruz |
| **CTIP2** | Rat monoclonal | Abcam |
| **CUX1** | Mouse monoclonal | Abnova |
| **EGFR1** | Mouse monoclonal | BD biosciences |
| **FOXG1** | Rabbit polyclonal | Abcam |
| **GAD65/67** | Rabbit polyclonal | Millipore |
| **HB9** | Mouse monoclonal | DHSB |
| **HOXB4** | Mouse monoclonal | DHSB |
| **MAP2** | Mouse monoclonal | Sigma |
| **NESTIN** | Mouse monoclonal | Millipore |
| **OLIG2** | Rabbit polyclonal | Millipore |
| **OTX2** | Goat polyclonal | R&D Systems |
| **p75** | Mouse monoclonal | Santa Cruz |
| **PAX6** | Mouse monoclonal | DHSB |
| **PSD-95** | Mouse monoclonal | Neuromab |
| **PSD-95** | Rabbit polyclonal | Cell Signaling |
| **REELIN** | Mouse monoclonal | Novus |
| **SATB2** | Mouse monoclonal | Abcam |
| **Sox1** | Rabbit polyclonal | Millipore |
| **SYNAPTOPHYSIN** | Rabbit polyclonal | Abcam |
| **SYNAPTOPHYSIN** | Mouse monoclonal | Millipore |
| **VGLUT1** | Mouse monoclonal | Neuromab |
| **β-3 Tub** | Mouse monoclonal | Sigma |
