# Supplementary material for: Physiological Normoxia and Absence of EGF Is Required for the Long-Term Propagation of Anterior Neural Precursors from Human Pluripotent Cells
Source: PLoS One. 2014 Jan 17;9(1):e85932. doi: 10.1371/journal.pone.0085932 (PMC3895023; doi:10.1371/journal.pone.0085932)
Supplement: Text S1 — Development of voltage-gated ion channel properties underlying the action potential. (DOCX) [file pone.0085932.s007.docx]

**Text S1:**

**Development of voltage-gated ion channel properties underlying the action potential.**

Rapidly-inactivating *Na_V_* currents (Fig. S4Ji) were isolated and measured from 3% O_2_ aNPC-derived neurons using a depolarising voltage-clamp protocol. The membrane potential was stepped in 10 mV increments (duration of 100 ms) from a holding potential of –74 mV. Subsequently, an equivalent protocol was repeated in the presence of TTX (300 nM) and current traces were subtracted from those recorded in the absence of TTX to yield the TTX-sensitive *Na_V_* - current. The mean normalised current-voltage (*I*-*V*) relationship for the *Na_V_* channels at Week 5 is shown in Fig S4J.ii. The developmental profile of *Na_V_* was monitored weekly (using a protocol where TTX-sensitive currents were activated and isolated using a step of +50 mV). From Weeks 1 to 5 *Na_V_* current densities increased significantly while the percentage of cells giving responses increased from 83.3 % to 100% (Fig. 5D). *Na_V_* current density reaches an apparent maximum level by Week 3, while the respective Week 4 and 5 data are not significantly different. Notable correlation can be observed between the development of *Na_V_* current density, the development of activity and AP properties.

The coordinated response of the AP relies also upon the functional expression of voltage-gated K^+^ (*K_V_*) channels of two varieties, *I_K_* and *I_A_*. *K_V_* channels importantly set the cellular *RMP*, AP firing threshold, repolarise the action potential and strongly influence firing properties, where *I_K_* and *I_A_* are important in repetitive firing and setting inter-spike interval, respectively[1]. *I_K_*-mediated currents were activated and isolated by a voltage activation protocol in the presence of TTX (300 nM), where cells were held at –54 mV and 10 mV incremental voltage pulses (250 ms) were applied and then repeated in the presence of *I_K_* inhibitor TEA (30 mM) to determine the non-*I_K_* component. The non-*I_K_* currents were subtracted from the former to determine the *I_K_*-specific current (current amplitudes were measured from the 200 ms time point). Responses were characterized by slow activation and sustained kinetics (delayed-rectifier) that gave an *I-V* plot characteristic of *I_K_* channels (Fig. S4 K.i and ii). The developmental maturation of the TEA-sensitive current was assessed over a 5-week period after differentiation and at all time points investigated, 100 % of neurons examined possessed an *I_K_* current response, which was assessed by measuring the TEA-sensitive current evoked by a voltage pulse of +60 mV. *I_K_* current density reaches maximum level by Week 3, but begins to decrease (significantly from Week 3 data) by Week 5 (Fig. 5D), which is consistent with maturing mammalian cortical neurons [2].

Distinctive transient *I_A_* currents were activated and isolated using a voltage-step protocol that gave a typical *I/V* plot as shown in Fig. S4 L.ii. *I_A_* channel activation was achieved using a protocol that initially stepped the holding potential from –74 mV to –114 mV (500 ms) after which the holding potential was increased in 10 mV increments (400 ms) before returning to –74 mV. The extracellular solution was additionally supplemented with Cd^2+^ (100 μM) to block VGGCs and TTX (300 nM). The non-specific *I_A_* component was isolated using stimulation protocol that takes advantage of the rapid de-activation properties of *I_A_*. In this regard, the protocol was identical to previous with the exception the neuron was initially ramped to –24 mV from –74 mV instead of -114 mV. The non-specific *I_A_* component was subtracted from the activation protocol to generate the *I_A_*-mediated current. The weekly development of the *I_A_* response was assessed by measuring the isolated current initially activated from a voltage ramp initially held at –114 mV (as described) then stepped 80 mV. A progressive increase in *I_A_* response was observed over the 5-week period where 100 % of neurons exhibit *I_A_* activity at week 5 from only 42.1 % at week 1. Strong (over 50-fold) development of *I_A_* current density significantly increases over the 5-week time period (Fig. 5D).

1. Hille B (2001) Ion Channels of Excitable Membranes. Sinauer Associates. 1 pp.

2. Picken Bahrey HL, Moody WJ (2003) Early development of voltage-gated ion currents and firing properties in neurons of the mouse cerebral cortex. J Neurophysiol 89: 1761–1773. doi:10.1152/jn.00972.2002.
